# Kaolin Particle Film Protects Grapevine cv. Cabernet Sauvignon Against Downy Mildew by Forming Particle Film at the Leaf Surface, Directly Acting on Sporangia and Inducing the Defense of the Plant

**DOI:** 10.3389/fpls.2021.796545

**Published:** 2022-01-10

**Authors:** Ying Wang, Xiao Cao, Yulei Han, Xing Han, Zhilei Wang, Tingting Xue, Qiuhong Ye, Liang Zhang, Xinyao Duan, Hua Wang, Hua Li

**Affiliations:** ^1^College of Enology, Northwest A&F University, Yangling, China; ^2^School of Food and Wine, Ningxia University, Yinchuan, China; ^3^Engineering Research Center for Viti-Viniculture, National Forestry and Grassland Administration, Yangling, China; ^4^Shaanxi Engineering Research Center for Viti-Viniculture, Yangling, China; ^5^China Wine Industry Technology Institute, Yinchuan, China

**Keywords:** Kaolin particle film, wine grape, resistance, plant protection, *Plasmopara viticola*

## Abstract

Downy mildew is a major threat to viticulture, leading to severe yield loss. The use of traditional copper-based fungicides is effective, but has adverse effects on the environment and human health, making it urgent to develop an environmentally friendly disease management program. Multi-functional kaolin particle film (KPF) is promising as an effective and safer treatment strategy, since this material lacks chemically active ingredients. In this study, ability of Kaolin particle film (KPF) pretreatment to protect grapevine leaves from *Plasmopara viticola* was tested and the mode of action of KPF was analyzed. KPF application reduced the disease severity and the development of intercellular hyphae. Additionally, there was reduced accumulation of H_2_O_2_ and malondialdehyde (MDA) with pretreatment. The observation of ultrastructure on the leaf surface showed KPF deposition and stomatal obstruction, indicating that KPF protected plants against disease by preventing the adhesion of pathogens to the leaf surface and blocking invasion through the stomata. KPF pretreatment also activated host defense responses, as evidenced by increased activities of anti-oxidative enzymes [superoxide dismutase (SOD), peroxidase (POD), and catalase (CAT)] and defense-related enzymes [phenylalanine ammonia-lyase (PAL), chitinases, and β-1,3-glucanases], increased phytohormone signals [abscisic acid (ABA), salicylic acid (SA), and jasmonic acid (JA)] and the up-regulation of defense genes related to plant defense. Overall, these results demonstrate that KPF treatment counters grapevine downy mildew by protecting leaves and enhancing plant defense responses.

## Introduction

There is extensive cultivation of wine grapes in China, with high economic, social, cultural, and ecological benefits (Zhang et al., 2021). However, the wet weather conditions of China favors disease, presenting cultivation challenges for the most widely grown variety, *Vitis vinifera*. Among oomycete-caused diseases, downy mildew is considered to be the most serious, resulting in severe yield losses (Gessler et al., 2011).

Grapevine downy mildew is caused by the biotrophic oomycete *Plasmopara viticola* [(Berk. and Curt.) Berl. and De Toni]. This pathogen infects the green organ of plants through the stoma, causing oilspots and the emergence of sporangiophores on the adaxial and abaxial leaf surfaces, respectively. At high humidity and in warm temperatures, over-wintered oospores can mature and germinate rapidly to form sporangia once exposed to water. Swarming zoospores released from sporangia reach stomata, where they encyst and then develop an intercellular mycelium. Sporangiophores are then then emerge through the stomatum and form sporangia, forming macroscopic mildew on the abaxial leaf surface (Burruano, 2000; Gessler et al., 2011).

When plants suffer from pathogen attack, stress conditions cause serious damage to plant cells. Once threat is recognized, plant cells react quickly, inducing plant defense responses, including generating reactive oxygen species, activating protein kinases, and expressing defense-related genes that produce pathogenesis-related (PR) proteins (Macho and Zipfel, 2014). First, plants respond to stress by the overproduction of reactive oxygen species (ROS) and malondialdehyde (MDA) (Yin et al., 2014), which can interrupt many biological functions, such as leads to membrane peroxidation, destroy membrane permeability, and membrane integrity, leading to cellular dysfunction and death (De Souza et al., 2014; Liu et al., 2015). ROS (mainly H_2_O_2_) production also act as secondary messengers to activate complex signaling cascades (Garcia-Brugger et al., 2006). Then, anti-oxidative enzymes, including superoxide dismutase (SOD), peroxidase (POD), and catalase (CAT) are activated to scavenge free radicals (Godard et al., 2009). In addition to reaction aimed at reducing damage, plant defense can be induced by complex cross-talk among salicylic acid (SA) (Loake and Grant, 2007; Pieterse et al., 2009), jasmonic acid (JA) (Hamiduzzaman et al., 2005), abscisic acid (ABA) (Allegre et al., 2009) and other plant hormones. Plant hormones and signaling molecules can also stimulate plant defense. For instance, SA activated grapevine resistance against downy mildew by inducing PR genes (Loake and Grant, 2007). Pathogenesis-related (PR) proteins such as chitinases (PR4) and β-1,3-glucanases (PR2), are usually considered to degrade the pathogen cell wall (Van Loon et al., 2006). In addition, secondary metabolites, which regulated by phenylalanine ammonia-lyase (PAL), also act to cope with pathogen attack, by host cell wall reinforcement and inhibition of pathogen growth (Underwood, 2012). However, these defense reactions are easier to be triggered in resistant grapevines, but in susceptible *V. vinifera*, these reactions may not be triggered therefore resulting in disease (Figueiredo et al., 2012; Yin et al., 2017). Therefore, there is a significant need for disease management strategies.

In addition to the breeding of disease-resistant varieties (Wang et al., 2021), there are many strategies for the control of grapevine downy mildew. For instance, β-1,3-glucan sulfated-laminarin (Trouvelot et al., 2008), chitosan (Aziz et al., 2006), non-protein amino acid BABA (Slaughter et al., 2008), and other plant extracts (Nesler et al., 2015; Krzyzaniak et al., 2018), have shown to be efficient elicitors of defense response in grapevine cells and plants for reducing *P. viticola* damage. As for biological control, *Fusarium proliferatum* G6 (Bakshi et al., 2001) and *Trichoderma harzianum* T39 (Perazzolli et al., 2011) have shown the effect of reducing disease development. However, the most effective treatment currently available is the use of fungicides such as Bordeaux mixture and other copper-based compounds. However, the long-term application of fungicides has resulted in extensive Cu accumulation in soil, presenting threats to the environment and human health (Komarek et al., 2010). Therefore, there is a need for alternative treatments that are effective but reduce the use of toxic chemicals.

Kaolin particle film (KPF) is an aqueous formulation from chemically inert mineral particles, which composed of aluminosilicate mineral (Al_4_Si_4_O_10_(OH)_8_), that has been shown to reduce insect and plant pathogen damage (Glenn et al., 1999; Puterka et al., 2000; Tubajika et al., 2007), mitigate summer stress (Dinis et al., 2020), enhance photosynthesis (Dinis et al., 2016b), and improve crop yields (Glenn and Puterka, 2010). Studies have shown that Kaolin suspension decrease leaf temperature and transpiration, increase leaf water potential, thereby improving the stomatal conductance and net photosynthesis (Shellie and King, 2013a; Dinis et al., 2018). In addition, Kaolin particle film application also has a stimulatory effect on the primary and secondary metabolism of grapevines, and improve the quality of berries (Conde et al., 2016, 2018; Dinis et al., 2016a). Our previous research has shown that KPF can be used on grapes to improve the levels of secondary metabolites, even in humid climate conditions (Wang et al., 2020). After spraying, the plant surface usually is covered with a hazy white layer, blocking pathogens and water from direct contact with the leaf surface (Walters, 2006). The unique benefit is that the application of KPF is environmentally friendly and there is low likelihood that insect pests and pathogens will develop resistance given the lack of chemically active components (Sharma et al., 2015). Therefore, KPF could be used as a long-term disease treatment option. However, except for only one study on cucumber downy mildew control by KPF (Haggag, 2002), there have been no studies of the use of KPF to as a potential control method for grapevine downy mildew.

In this study, the ability of KPF to protect grapevine leaves against downy mildew was assessed and its mode of action was determined. First, the efficacy of KPF application against downy mildew was evaluated on leaf discs under artificial inoculation conditions. Next, the ultrastructure in cells and on the leaf surface was observed to better understanding the mode of action of KPF. The effect of KPF to stimulate plant defense was investigated by analyzing plant defense responses in grapevine leaves. Finally, the effect of KPF to zoospore release was observed to understanding the direct effect of Kaolin on *P. viticola* sporangia.

## Materials and Methods

### Pathogens

Pathogen samples were collected in the Grape Repository of Northwest A&F University, Yangling, Shaanxi, China. During summer in 2020, leaves presenting oily spot symptoms were harvested and were placed, with the abaxial surface facing up, in the dark at 100% relative humidity (RH) for 24 h. Sporangia were collected from the abaxial surfaces of grapevine leaves with freshly sporulating lesions using a soft brush and suspended in sterile water. The sporangial suspensions were counted with a hemocytometer under a light microscope and then adjusted to a concentration of 8 × 10^5^ sporangia.mL^–1^ before being used immediately.

### Kaolin Particle Film

Kaolin particle film is based on kaolin, a white, non-porous, fine-grained, chemically inert aluminosilicate mineral (Al_4_Si_4_O_10_(OH)_8_). The KPF used in this study was developed by scientists at the College of Enology of Northwest A&F University, and produced by the Research and Development Center of Biorational Pesticide. KPF is powder-like and disperses easily in water. After mixing with water at the desired proportion, KPF can be directly sprayed on the plant surface for complete and even coverage using a conventional sprayer (Shellie and King, 2013b). After application and drying, the KPF will appear hazy white on plant surfaces.

### *Plasmopara viticola* Inoculation and Treatment

Healthy leaves from *Vitis vinifera* L. cv. Cabernet Sauvignon were obtained from the Grape Repository of Northwest A&F University, Yangling, Shaanxi, China. Leaves at the third and fourth positions from the apex were collected from grapevines cultivated in a vineyard, surface-sterilized with bleach (0.01%), and then washed three times with sterile water. Leaf discs of 1.4 cm in diameter were punched from leaves using a corkborer. The discs were mixed thoroughly and placed (bottom side up) on petri dishes containing a wet filter paper. All discs were divided into four groups and pre-treated with different KPF doses using a manual hand sprayer. The discs were then inoculated over their abaxial surfaces with five 20 μl droplets of sporangial suspension (8 × 10^5^ sporangia mL^–1^). Petri dishes with inoculated discs (adaxial surface downwards) were incubated in a growth chamber at 20 ± 2°C and 100% RH for 24 h in the dark, and then under a 16 h light and 8 h dark photoperiod for an additional 8 days (Liu et al., 2015). The inoculum droplets on leaf discs were removed after 24 h. The four treatments were as follows: treatment with 1% (w/v) KPF + *P. viticola* inoculation (1% KPF); treatment with 2% (w/v) KPF + *P. viticola* inoculation (2% KPF); treatment with 3% (w/v) KPF + *P. viticola* inoculation (3%KPF); (4) distilled water + *P. viticola* inoculation (Control). This experiment was conducted as a completely randomized design.

Three independent experiments with three biological repetitions each were carried out in this study. For each independent experiment, 150 leaf discs per treatment were randomly allocated to 30 petri dishes, 5 leaf discs in each petri dish. A total of 30 leaf discs in 6 petri dishes were randomly selected for sporulation symptoms observation at 1, 3, 5, 7, and 9 days post- inoculation (dpi) respectively. 30 discs were used as one biological replicate, and three biological repetitions were carried out for an independent experiment. Discs for the same biological repetition were collected from same plant. Discs for three different biological repetitions were collected from three different palnts. Same inoculum was used for each independent experiment at the same day. After sporulation symptoms observation, same discs were used for investigation of sporangial production. Then samples were quickly frozen in liquid nitrogen and stored at −80°C. Then the same independent experiment was carried out three times, using different batch of inoculum on a different day. And discs for different independent experiment were collected from different plants.

Then the same independent experiment was repeated for the visualization of *P. viticola* in leaf tissues and stomata. Samples of infected stomata (7-10 discs each treatment) were collected for observation by scanning electron microscope at 9 dpi and processed immediately. 30 discs for each treatment were collected at 12, 24, 48, 72, and 96 h post-inoculation (hpi) for fluorescence microscopic visualization of *P. viticola* in leaf tissues and were processed immediately. The petri dishes in the incubators for observation and storage were selected at random.

### Disease Incidence and Severity

Discs of each treatment were inoculated with five 20 μL droplets of the sporangial suspension and incubated as described above. Quantitative symptoms of infection such as disease index (DI) were visually analyzed at 1, 3, 5, 7, and 9 days post-inoculation (dpi). To score disease severity, the percentages of leaf disc areas exhibiting signs of sporulation (visible white downy mildew on leaf surface composed of sporangiophores and sporangia) were measured with Photoshop CS5 software. The leaf discs were classified according to the Desaymard “0-10” classification method (Li, 1991), where 0 = absence of sporulation, 1 = 0.1 − 2.5%, 2 = 2.6 − 5.0%, 3 = 5.1 − 15.0%, 4 = 15.1 − 30.0%, 5 = 30.1 − 50.0%, 6 = 50.1 − 70.0%, 7 = 70.1 − 85.0%, 8 = 85.1 − 95.0%, 9 = 95.1 − 97.5%, and 10 = 97.6 − 100% of the leaf area covered by sporulation. The DI was calculated using the following formula:


$$\mathrm{DI}=\frac{\sum \left(\mathrm{rank}\times \mathrm{numbers}\mathrm{disk}\mathrm{leaf}\mathrm{infected}\mathrm{in}\mathrm{that}\mathrm{rank}\right)}{\mathrm{Total}\mathrm{disc}\mathrm{numbers}\times \mathrm{highest}\mathrm{rank}}\times 100$$

Disease severity was assessed as the area under the disease progress curve (AUDPC). The AUDPC was calculated between 1 and 9 dpi. To plot the disease progress curve, evaluations were performed at 1, 3, 5, 7, and 9 dpi and the following equation (Campbell and Madden, 1990) was used:


$$AUDPC=\sum_{i}^{n-1}\left[\left(\frac{y_{i}+y_{i+1}}{2}\right)\left(t_{i+1}-t_{i}\right)\right]$$

In this equation, n is the total number of assessments, t_*i*_ is the time at which evaluation i was performed, and y_*i*_ is the DI at time t_*i*_.

Percentage of protection achieved by a given treatment of various doses of KPF was calculated by comparing the AUDPC, on that particular treatment with that of the non-treated control in the same trail according to the following equation, where AUDPC_*Control*_ = AUDPC of the non-treated control and AUDPC_*KPF*_ = AUDPC of a KPF treatment.


$$\%\mathrm{Protection}=\frac{{\mathrm{AUDPC}}_{\mathrm{Control}}-{\mathrm{AUDPC}}_{\mathrm{KPF}}}{{\mathrm{AUDPC}}_{\mathrm{Control}}}\times 100$$

### Determination of Zoospore Release and Sporulation

For investigation of sporangial production over infection period, the number of sporangia produced per cm^2^ was calculated and used to scale the daily production of sporangia in the detached leaf trial. For each treatment, 30 discs with sporulation were collected randomly every other day, and agitated for 5 min in 2 mL of distilled water. The number of sporangia mL^–1^ of the resulting suspension was estimated with a hemocytometer under the optical microscope. A total of 10 hemocytometer chambers were examined for each suspension. The number of sporangia mL^–1^ on 9 dpi of the control group was used as the reference to calculate the daily cumulative proportion of sporangia (DCP). The sporangia per cm^2^ leaf area were calculated by dividing the area of the selected discs at 9 dpi. Three independent biological repetitions were performed.

The effect of KPF on zoospore release for *P. viticola* was evaluated under controlled conditions. The four treatments were as follows: (1) 0.1% (w/v) KPF + *P. viticola* (0.1% KPF); (2) 0.25% (w/v) KPF + *P. viticola* (0.25% KPF); (3) 0.5% (w/v) KPF + *P. viticola* (0.5%KPF); (4) distilled water + *P. viticola* (Control). More than 0.50% of KPF seriously affected the observation of sporangia, due to excessive kaolin particles. The sporangial suspensions was centrifuged at 8,000 rpm for 10 min, then were adjusted to 1.6 × 10^6^ sporangia.mL^–1^, which was twice the initial concentration. The KPF suspensions concentration was configured as 0.20%, 0.50%, and 1.00%. Then the sporangial suspensions and KPF suspensions were mixed in a ratio of 1:1. In this way, the concentration of KPF is adjusted to 0.10%, 0.25%, and 0.50%. The Control treatment was treated with equivalent sterile water. Then the mixture was placed in dark at 100% RH. Then, zoospore release was estimated based on the number of empty sporangia (sporangia that had released their zoospores) under the optical microscope (Caffi et al., 2016) at 0, 2, 4, 6, 12, 24, 48, and 72 h. A total of 10 chambers were examined for each suspension. This experiment was conducted as a completely randomized design with three biological replicates.

### Fluorescence Microscopic Visualization of *Plasmopara viticola* in Leaf Tissues

Samples of leaf discs were collected at 12, 24, 48, 72, and 96 hpi for observation of intercellular infection structures 30 discs for each treatment were collected for image analysis per sampling time. Then, discs were stained with KOH-aniline blue as described previously with slight modification (Hood and Shew, 1996; Diez-Navajas et al., 2007). Briefly, 1 M KOH was added to leaf discs and the mixing systems were placed in a water bath at 100°C for 15 min. The KOH was then poured out and the discs were washed three times with distilled water, each time for 15-20 min. After washing, the discs were stained with 0.05% aniline blue in 0.067 M K_2_HPO_4_ (pH 9-10) and kept away from light. For microscopic observation, the samples were examined under blue/violet light with a fluorescence microscope (LECIA DM6 B, Germany).

### Evaluation of Oxidative Stress Indices

Determination of hydrogen peroxide (H_2_O_2_) in plant extracts was performed using titanium (IV) (Patterson et al., 1984). Samples of discs (0.5 g) were ground in liquid nitrogen, extracted with 7 mL of 5% (w/v) trichloroacetic acid, and then centrifuged at 10,000 rpm for 20 min. The supernatants were assayed for H_2_O_2_.

Malondialdehyde (MDA) content was determined by measuring the thiobarbituric acid-reactive substances (Hodges et al., 1999). To do this, disc samples (2.0 g) were homogenized in 15 mL 0.1% TCA and then centrifuged at 5,000 rpm for 10 min. Five milliliters of 5% TCA containing 0.5% TBA were added to 1 mL of the supernatant and then incubated in boiling water for 10 min. The reaction tubes were then transferred to ice water to stop the reaction. MDA absorption was measured spectrophotometrically at 450, 532, and 600 nm. Three biological replicates of the experiment were performed, and all data were analyzed in triplicate.

### Scanning Electron Microscopic Observation of Infected Stomata

A 7-10 discs for each treatment were collected for image analysis at 9 dpi and washed in distilled water to remove kaolin deposits and impurities on the leaf disc surface. Samples from the middle part of the leaf were cut into 5 × 5 mm pieces to ensure sample uniformity. At least three replicates were used for each treatment. To leaf samples, 4% glutaraldehyde was added and incubated at 4°C overnight. The next day, the small pieces were washed four times, 10 min each, with 0.1 M phosphate-buffered saline (pH 6.8). The fragments were then dehydrated in a graded series of aqueous ethanol solutions (30, 50, 70, 80, and 90% ethanol), each step for 15-20 min, and then three washes with 100% ethanol for 30 min each time. Finally, samples were critical point dried in CO_2_ and then coated with gold. Samples were placed bottom-side up and viewed with a Nano SEM-450 (FEI, United States) scanning electron microscope at 10 kV. All the collected discs were observed, and four representative illustrations from different independent discs were selected to show.

### Evaluation of Enzymes Related to Plant Defense

Samples of leaf discs (1.0 g) from all treatment groups were ground in a chilled mortar with 1% (w/v) polyvinylpolypyrrolidone, homogenized with 15 mL of 50 mM potassium phosphate buffer (pH 7.8), and then centrifuged at 10,000 rpm for 15 min. The resulting supernatant was used for the determination of antioxidant enzyme activities. Superoxide dismutase (SOD; EC 1.15.1.1) activity was estimated by NBT (Giannopolitis and Ries, 1977); catalase (CAT; EC 1.11.1.6) activity was evaluated according to the decomposition rate of hydrogen peroxide (Aebi, 1984); peroxidase (POD; EC 1.11.1.7) activity was measured by guaiacol (Rao et al., 1996). The activities of these enzymes were expressed as U•g^–1^FW•h^–1^. Three biological replicates were performed, and all parameters were analyzed in triplicate.

The activity of phenylalanine deaminase (PAL) was determined by detecting the amount of generated trans-cinnamic acid (Meyer et al., 1999). Leaf samples (2.0 g) were homogenized in a chilled mortar with 15 mL of 0.1 M boric acid buffer (pH 8.8) containing 5 mM mercaptoethanol, 2 mM EDTA, and 40 g polyvinylpolypyrrolidone, with quartz sand added for efficient homogenization. The homogenate was centrifuged at 12,000 rpm for 30 min at 4°C. Enzyme activity was determined using a reaction system that included 0.5 mL of supernatant, 0.5 mL of 20 mM L-phenylalanine, and 3 mL of 50 mM boric acid buffer (pH 8.8). The mixed solution was incubated in a water bath (constant 37°C) for 60 min and then 0.1 mL of 6 M HCl was added to stop the reaction. Absorbance was then determined at 290 nm. The PAL activity was expressed as U•g^–1^FW•h^–1^.

The activity of β-1,3-glucanase was determined according to the amount of generated hydrolyzed sugars (Denault et al., 1978). Disc samples (0.5 g) were homogenized in a chilled mortar with 5 mL of 0.1 M sodium acetate buffer (pH 5.2) containing 5 mM mercaptoethanol, 1 M EDTA, and 1 g L-ascorbic acid, with quartz sand added for efficient homogenization. The reaction mixture contained 100 μL 4 g•L^–1^ laminarin and 100 μL enzyme extract. The mixture was incubated at 37°C for 40 min and the reducing ends of the hydrolyzed sugars were determined by DNS (Dygert et al., 1965). The β-1,3-glucanase activity was expressed as U•g^–1^FW•h^–1^.

Chitinase (EC 3.2.1.14) was assessed based on generated N-acetyl glucose and detected with a chitinase assay kit according to the manufacturer’s instructions (Cat#BC0820, Solarbio, China). The chitinase activity was expressed as U•g^–1^FW•h^–1^. Three biological replicates were performed, and all parameters were analyzed in triplicate.

### Evaluation of Endogenous Plant Hormones

Plant hormones were assessed by enzyme-linked immunosorbent assays (ELISA). Briefly, a 0.5 g disc sample was weighted and ground into powder in liquid nitrogen. The powder was then homogenized in a chilled mortar with 4 mL of 80% methanol containing 1 mM BHT (dibutyl hydroxyl-toluene). The mixture was incubated at 4°C for 4 h and then centrifuged at 3,500 rpm for 8 min at 4°C. The precipitate was re-extracted one time, and the supernatant was passed through a Truserco^®^ C18 solid phase extraction column (Casco, China). The liquid was quickly evaporated, and 1 mL of 50 mM phosphate buffer saline (pH = 7.5) with Tween and gelatin was added. This solution was then used for hormone determination.

Abscisic acid (ABA) in each sample was assessed by ELISA [ml077235 Plant Abscisic Acid (ABA) ELISA Kit, mlbio, China]; salicylic acid (SA) was assessed by ELISA [DECO5174 Plant Salicylic Acid (SA) ELISA Kit, DECO, China]; jasmonic acid (JA) was assessed by ELISA [DECO5172 Plant Jasmonic Acid (JA) ELISA Kit, DECO, China]. All measurements were carried out following the manufacturer’s instructions. The OD values were measured using a Victor X3 microplate reader (PerkinElmer, United States). Three biological replicates were performed, and all parameters were analyzed in triplicate.

### RNA Extraction and Transcriptional Analysis by Real-Time qPCR

Samples of 100 mg of grape leaf tissue previously ground in liquid nitrogen were used for total RNA extraction. Three samples were used for each measurement of total RNA. Total RNA was extracted from each sample using the E.Z.N.A.^®^ Plant RNA Kit (Omega, United States), following the manufacturer’s instructions. Next, cDNA was synthesized from 1 μg of total RNA using the First-strand cDNA Fast Synthesis Kit (Cofitt, China). Real-time PCR analysis was then performed with ChamQ Universal SYBR qPCR Master Mix (Vazyme, China) using 2 μL cDNA (diluted 1:5 in ddH_2_O) in a final reaction volume of 20 μL per well. The qPCR reactions were conducted on a qTOWER3 G (Analytik Jena, Germany).

Gene selected for further analysis included *PAL* (phenylalanine ammonia-lyase), *ICS2* (isochorismate synthase 2), *PAD4* (phytoalexin deficient 4), *EDS1* (enhanced disease susceptibility 1), *NPR1* (non-expresser of pathogenesis related gene 1), *TGA1* (transcription factor TGA1), *PR1* (pathogenesis-related protein 1), *PR2* (beta 1-3 glucanase), and *PR4* (pathogenesis-related protein 4). Actin was used as the internal control (Kim et al., 2003). Gene-specific primer pairs used for each target or reference gene are listed in Supplementary Table S1 (Conde et al., 2015; Cappelletti et al., 2016; Liu et al., 2016). Melting curve analysis was performed for specific gene amplification confirmation. The expression values were normalized by the average of the expression levels of the reference genes and calculated using the 2^–Δ^ ^Δ^ *^Ct^* method (Livak and Schmittgen, 2001). Three biological replicates were performed, and all parameters were analyzed in triplicate.

### Statistical Analysis

All data were analyzed and graphed using the GraphPad Prism software. Values are presented mean ± standard interval of three independent experiments, with three biological repetitions, with three technical repetitions each. The AUDPC was evaluated by one-way ANOVA. Other data were evaluated by two-way ANOVA. Tuckey’s test was carried out among the different treatments for each time point, and among different time point for each treatment, where differences were considered significant at *P* ≤ 0.05.

## Results

### Severity of Downy Mildew Infection

Leaf discs were pretreated with KPF at different doses and then inoculated with *P. viticola* sporangia. The leaf area covered with pathogen sporulation symptoms was evident on the discs at 3 dpi. The different KPF treatments of leaf discs showed different levels of protection to the inoculated *P. viticola*.

**FIGURE 1:**
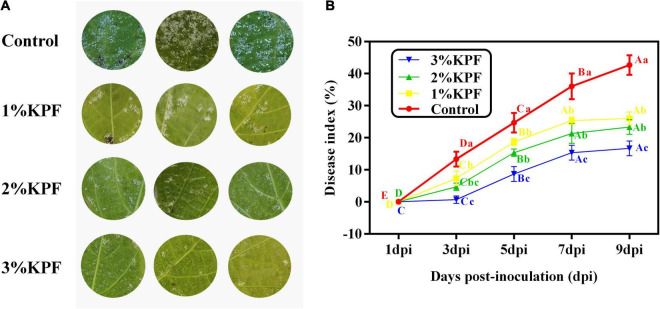
Effect of KPF on downy mildew development in grapevine leaf discs. *V. vinifera* (cv. Cabernet Sauvignon) leaf discs were treated with sterile water (Control), 1%, 2%, and 3% KPF and then were inoculated with a fresh *P. viticola* sporangia suspension (8 × 10^5^ sporangia.ml^– 1^). The leaf area covered with pathogen sporulation symptoms for each disc was determined and 30 discs per biological replicated was used for disease index (DI) calculation at 1, 3, 5, 7, and 9 dpi. Three independent experiments with three biological repetitions each were carried out. Results correspond to the mean ± standard interval of three independent experiments, with three biological repetitions, with three technical repetitions each. According to Tukey’s test (*P* ≤ 0.05), means with the same lowercase letter are not significantly different among the different treatments for each time point, and means with the same uppercase letter are not significantly different among the different time points for each treatment. **(A)** Effect of KPF on downy mildew development in grapevine leaf discs at 9 dpi; **(B)** Disease progress curve. The curve was profiled according to the DI.

Hyphae on discs were photographed at 9 dpi. As shown in Figure 1A, although necroses were localized, extensive sporulation was observed at 9 dpi in discs of the control group. In this group, about 60% of the areas of the leaf discs showed signs of *P. viticola* infection. In contrast, the KPF-treated discs showed slower and lighter development of hyphae than the control group, with localized necroses. The disease index is a comprehensive indicator of plant severity. Figure 1B show the disease index were gradually reduced with increased KPF dose. The maximal protection level, as evidenced by significant inhibition of *P. viticola* sporulation symptoms, was obtained at 3% KPF concentration. AUDPC analysis also showed that the KPF affected the severity of leaf spot (Table 1). Application of KPF decreased AUDPC significantly. 31.99, 44.33, and 65.74% protection were achieved from 1, 2 and 3%KPF treatment, respectively.

**TABLE 1:** Effect of Kaolin particle film application on grapevine downy mildew development.

Treatment	AUDPC^a^	% Protection^b^
Control^c^	264.67 ± 7.57a^d^	
1%KPF	180.00 ± 7.21b	31.99 ± 2.72a
2%KPF	147.33 ± 9.87c	44.33 ± 3.73b
3%KPF	90.67 ± 5.03d	65.74 ± 1.90c

*^a^AUDPC (area under the disease progress curve) was calculated from diease severity that was rated from 1 to 9 dpi intervals.*

^b^%Protection = [(AUDPC_Control_ – AUDPC_KPF_)/AUDPC_Control_]*100 where AUDPC_Control_ = AUDPC of the non-treated control and AUDPC_KPF_ = AUDPC of a KPF pretreatment.

*^c^Control, the non-treated control was sprayed with KPF equal dose deionized water.*

*^d^Three independent experiments with three biological repetitions each were carried out. Results correspond to the mean ± standard interval of three independent experiments, with three biological repetitions, with three technical repetitions each. Means with the same letter are not significantly different according to Tukey’s test (P ≤ 0.05).*

### Development of *Plasmopara viticola* Colonization in Leaf Tissue

**FIGURE 2:**
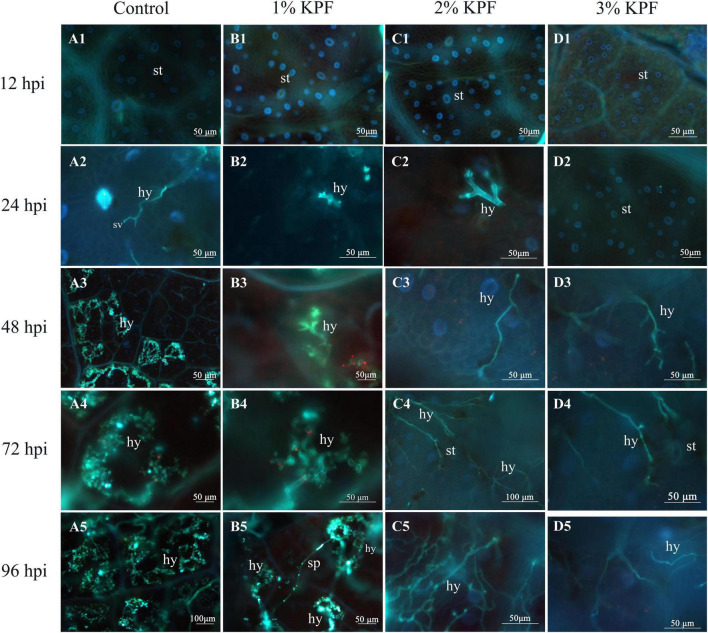
Fluorescence microscope visualization of *P. viticola* in leaf discs at 12, 24, 48, 72, and 96 dpi. *V. vinifera* (cv. Cabernet Sauvignon) leave discs were treated with sterile water (Control **A1–A5**), 1% KPF **(B1–B5)**, 2% KPF **(C1–C5)**, 3% KPF **(D1–D5)** and then were inoculated with a fresh *P. viticola* sporangia suspension (8 × 10^5^ sporangia.ml^– 1^). Samples of leaf discs were collected at 12, 24, 48, 72, and 96 hpi for observation of intercellular infection structures, 30 discs for each treatment were collected for image analysis per sampling time. *st*, stomatum; *sv*, substomal vesicle; *hy*, hyphae; *sp*, sporangiophore.

The development of *P. viticola* was compared for leaves subjected to different doses of KPF treatment by fluorescence microscopy. At 12 hpi, the invasion of *P. viticola* was not observed in any treatment (Figures 2A1-D1). At 24 hpi, the substomatal vesicle originating from the infection peg was visible in the control group leaves (Figure 2A2), and in a few infected areas, the primary hypha was also observed (Figures 2B2,C2). In the 3% KPF treated leaves, hypha were not observed until 48 hpi (Figure 2D3). By 24 hpi, there were significant differences among the treatments in the colonization of *P. viticola*. In the control and 1% KPF groups, there were primary hyphae inside the leaf tissues, and significantly restricted mycelium growth in 2% and 3% KPF treated leaves (Figures 2A3-D3). By 72 hpi, in the control group, the *P. viticola* started to expand rapidly inside the infected tissues (Figure 2A4). Compared to the control, the mycelium growth in the 1% KPF treatment was much slower, and the pathogen growth was still very restricted in 2% and 3% KPF treatments (Figures 2B4-D4). By 96 hpi, the mycelium growth in the control had increased. Abundant fluorescence was observed in the control and 1% KPF treatments, indicating successful sporulation and developed sporangiophores (Figures 2A5,B5). However, no sporangiophores were observed in the 3% KPF treatment (Figure 2D5). Compared to the control, KPF-treated leaves showed lower hypha densities and slower development of *P. viticola* in intercellular tissues.

### Oxidative Damage of Leaf Discs

**FIGURE 3:**
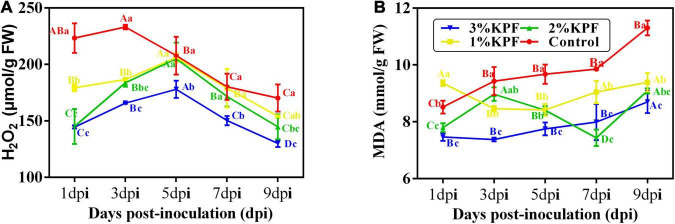
Effect of KPF pretreatment on oxidative stress indices. 30 discs per biological replicate were collected at 1, 3, 5, 7, and 9 dpi. Three independent experiments with three biological repetitions each were carried out. Results correspond to the mean ± standard interval of three experiments, with three independent biological repetitions, with three technical repetitions each. According to Tukey’s test (*P* ≤ 0.05), means with the same lowercase letter are not significantly different among the different treatments for each time point, and means with the same uppercase letter are not significantly different among the different time points for each treatment. **(A)** Hydrogen peroxide (H_2_O_2_); **(B)** Malondialdehyde (MDA).

During this observation period, the amount of H_2_O_2_ increased first and then decreased. The overall H_2_O_2_ content was lower in KPF-treated leaves than in control, and the contents were gradually reduced by increased KPF dose (Figure 3A). The MDA content increased with infection progress except for at a few points (Figure 3B). Leaves treated with KPF maintained lower MDA during the infection period.

### Ultrastructure of Stomata With *Plasmopara viticola* Infection

**FIGURE 4:**
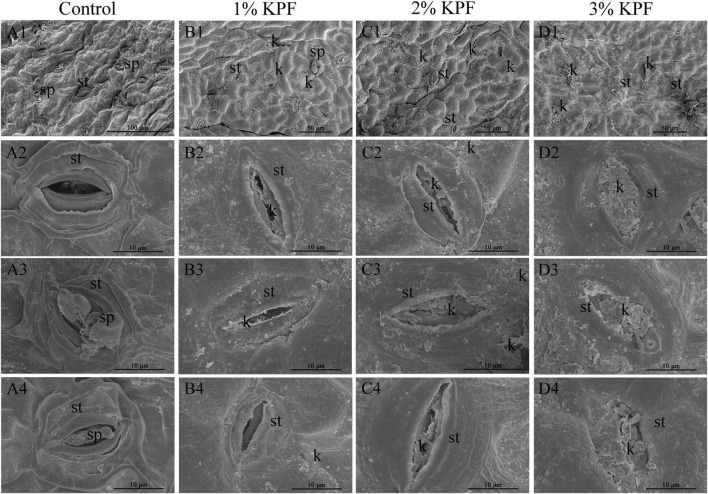
Scanning electron microscope observation of infected stomata of leaf discs at 9 dpi. *V. vinifera* (cv. Cabernet Sauvignon) leaf discs were treated with **(A1–4)** sterile water (Control), **(B1–4)** 1%, **(C1–4)** 2%, **(D1–4)** 3% KPF and then were inoculated with a fresh *P. viticola* sporangia suspension (8 × 105 sporangia.ml^−1^). 7-10 discs for each treatment were collected for image analysis at 9 dpi. Four representative illustrations from different independent discs were selected to show. *k*, KPF deposition; *st*, stomatum; *sp*, sporangiophore.

Scanning electron microscopy (SEM) observation showed that spray application of KPF left deposits on leaf surface and stomata. In control leaf discs, the stomata were well-opened (Figures 4A1,A2) and full sporangiophores developed (Figures 4A1,A3,A4), demonstrating successful infection. Conversely, KPF-treated leaves showed the presence of deposits with stomata that were deposited by the kaolin deposits to varying degrees (Figures 4B1-B4,C1-C4,D1-D4). In 1% KPF treatment, there was less kaolin deposited on the discs. For the most concentrated treatment (3% KPF), there was a lot of kaolin deposited on the leaves and the stomata were almost completely plugged even after washing the discs.

### Activity of Antioxidant and Disease-Responsive Enzyme in Leaves

**FIGURE 5:**
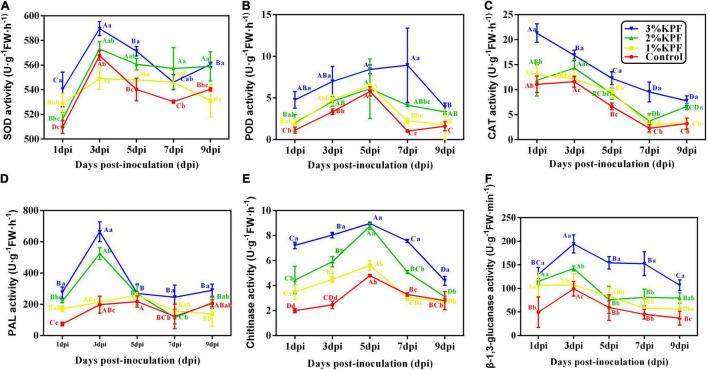
Effect of KPF pretreatment on the activities of enzymes related to plant defenses. 30 discs per biological replicate were collected at 1, 3, 5, 7, and 9 dpi. Three independent experiments with three biological repetitions each were carried out. Results correspond to the mean ± standard interval of three experiments, with three independent biological repetitions, with three technical repetitions each. According to Tukey’s test (*P* ≤ 0.05), means with the same lowercase letter are not significantly different among the different treatments for each time point, and means with the same uppercase letter are not significantly different among the different time points for each treatment. **(A)** superoxide dismutase (SOD) activity; **(B)** peroxidase (POD) activity; **(C)** catalase (CAT) activity; **(D)** phenylalanine ammonia-lyase (PAL) activity; **(E)** Chitinase activity; **(F)** β-1, 3-glucanase activity.

The main antioxidant enzymes in plants are SOD, POD, and CAT. The activities of SOD and POD were induced after leaves were infected, and then decreased, with a gradual decrease in CAT during the infection period (Figures 5A-C). The maximal activities among enzymes occurred at different times after infection. The maximum CAT activity occurred at the beginning of infection and then decreased (Figure 5C). The SOD activity increased rapidly and peaked at 3 dpi (Figure 5A), while POD activity increased slowly. The maximum POD activity for the 3% KPF treatment occurred at 7 dpi, with the highest for other treatments at 5 dpi (Figure 5B). SOD activity decreased slowly from maximal levels, with more dramatic decreases in POD and CAT activities. KPF application changed the activity of all three enzymes. During the infection period, the activities of SOD, POD, and CAT were generally higher in KPF-treated leaves than in those from the control group, especially for the 3% KPF treatment.

Phenylalanine ammonia-lyase (PAL), chitinase, and β-1, 3-glucanase are important enzymes closely associated with resistance to plant disease and were activated upon inoculation (Figures 5D-F). PAL activity peaked at 3 dpi in 3% and 2% KPF treated leaves, but peaked at 5 dpi in the control and 1% KPF treated leaves. The PAL activity then decreased and was maintained at a low level. The 2% and 3% KPF treatment significantly increased PAL activity (Figure 5D). β-1,3-glucanase and chitinase activity improved and decreased slowly. The highest level of chitinase was observed at 5 dpi in all treatments. The chitinase activity was gradually increased by increased KPF dose (Figure 5E). β-1, 3-glucanase activity peaked at 3 dpi and then decreased slowly. The 3% KPF treatment improved the β-1, 3-glucanase activity significantly, without significant overall differences among the control, 1% KPF, and 2% KPF treatments (Figure 5F).

### Phytohormone Levels in Leaves

**FIGURE 6:**
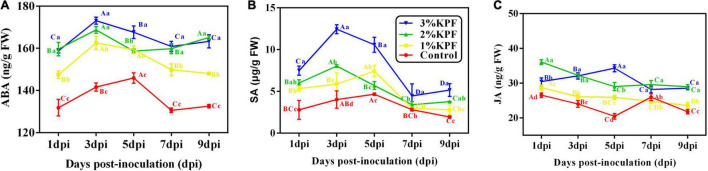
Effect of KPF pretreatment on levels of endogenous plant hormone. 30 discs per biological replicate were collected at 1, 3, 5, 7, and 9 dpi. Three independent experiments with three biological repetitions each were carried out. Results correspond to the mean ± standard interval of three independent experiments, with three biological repetitions, with three technical repetitions each. According to Tukey’s test (*P* ≤ 0.05), means with the same lowercase letter are not significantly different among the different treatments for each time point, and means with the same uppercase letter are not significantly different among the different time points for each treatment. **(A)** ABA; **(B)** SA; **(C)** JA.

As shown in Figure 6, the content of ABA and SA changed significantly in response to *P. viticola* infection, with all tested phytohormones improved by KPF treatment (Figure 6). After inoculation with *P. viticola*, the ABA and SA content first increased and then decreased. ABA content peaked at 3 dpi in KPF pretreated leaves, but peaked at 5 dpi in control leaves. However, the ABA content in 3% and 1% KPF treated leaves declined slower than that in the control (Figure 6A). In response to inoculation, the SA level of the control plants increased slowly at first, reached a peak at 5 dpi, and then decreased (Figure 6B). In the 2% and 3% KPF groups, the SA content increased rapidly to a maximum at 3 dpi, decreased at 5 and 7 dpi, and then showed a slight increase at 9 dpi. Compared to the control, there was significantly increased SA in the 1% KPF treatment, though this improvement was slower and later than that seen for the 2% and 3% KPF treatments. The content of JA decreased slowly during *P. viticola* infection (Figure 6C). There were small increases in the levels of JA for KPF relative to the control, but little effect on the downward trend during infection.

### Expression of Key Genes in the SAR Pathway

**FIGURE 7:**
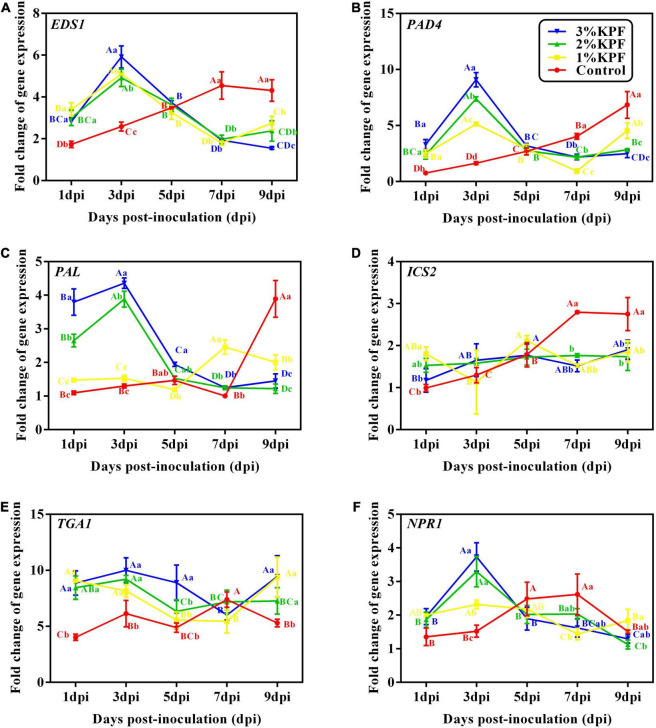
Effect of KPF pretreatment on leaf discs relative expression of defense-related genes. Data represent the fold-change in gene expression in *P. viticola* inoculated leaf discs vs. healthy leave discs. 30 discs per biological replicate were collected at 1, 3, 5, 7, and 9 dpi. Three independent experiments with three biological repetitions each were carried out. Results correspond to the mean ± standard interval of three independent experiments, with three biological repetitions, with three technical repetitions each. According to Tukey’s test (*P* ≤ 0.05), means with the same lowercase letter are not significantly different among the different treatments for each time point, and means with the same uppercase letter are not significantly different among the different time points for each treatment. **(A)** *EDS1*; **(B)** *PAD4*; **(C)** *PAL*; **(D)** *ICS2*; **(E)***TGA1*; **(F)** *NPR1*.

Salicylic acid signal pathway activators, *EDS1* and *PAD4* are induced before SA accumulation, and exhibited similar expression patterns after *P. viticola* inoculation among the different KPF treatments (Figures 7A,B). In the control group, the expression of *EDS1* and *PAD4* showed slight changes in response to the pathogen in an early stage, and increased slowly during infection. In the KPF treatment groups, *EDS1* and *PAD4* were sharply induced in the early stage of infection, peaking at 3 dpi followed by a decrease in expression level. The transcription of *EDS1* was similar among the three KPF treatments, and the maximum was up-regulated by about 5-fold (Figure 7A). The maximum expression of *PAD4* was gradually increased by increased KPF dose (Figure 7B).

The two genes, *PAL* and *ICS2*, that modulate SA synthesis, exhibited different expression patterns after *P. viticola* inoculation among the different KPF treatments (Figures 7C,D). *PAL* was activated slightly in the control until 9 dpi, but was greatly up-regulated in 2% and 3% KPF treatments and reached a peak of about 4-fold at 3 dpi, followed by a sharp decline (Figure 7C). The transcription of *PAL* was also increased by 1% KPF at later and lower levels. The expression of *ICS2* in control increased with inoculation, indicating activation by *P. viticola* (Figure 7D). The KPF treatments showed no significant effect on *ICS2* expression.

The two genes (*TGA1* and *NPR1*) regulated by SA were activated by KPF (Figures 7E,F). *NPR1* showed an immediate response to *P. viticola* infection in the control group, with a continuous increase to about 2-fold at 7 dpi and then a decrease (Figure 7F). NPR1 was activated by KPF treatment significantly at 3 dpi, then decreased to a lower level at 5 dpi that was maintained. The *TGA1* transcription factor gene showed a rapid response to *P. viticola* infection, with increased expression at the beginning of inoculation in all treatments (Figure 7E). In the control group, the expression of *TGA1* increased slightly during infection. KPF treatment increased the expression of *TGA1* significantly by about 10-fold. The expression then declined slowly, followed by a slight increase.

**FIGURE 8:**
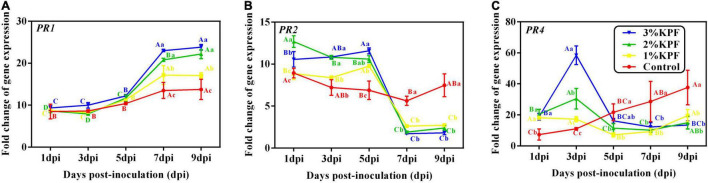
Effect of KPF pretreatment on leaf discs relative expression of pathogenesis-related protein gene. Data represent the fold-change in gene expression in *P. viticola* inoculated leaf discs vs. healthy leaf discs. Three independent experiments with three biological repetitions each were carried out. Results correspond to the mean ± standard interval of three independent experiments, with three biological repetitions, with three technical repetitions each. According to Tukey’s test (*P* ≤ 0.05), means with the same lowercase letter are not significantly different among the different treatments for each time point, and means with the same uppercase letter are not significantly different among the different time points for each treatment. **(A)** *PR1*; **(B)** *PR2*; **(C)** *PR4*.

KPF treatments induced much higher expression of *PR* genes compared to the levels in the control (Figure 8). Expression of the *PR1* gene was induced by *P. viticola* during infection (Figure 8A). KPF increased the expression of *PR1* at 7 dpi and 9 dpi in a dose-dependent manner (20-fold increase by 2% and 3% KPF and 15-fold increase by 1% KPF). *PR2* gene was activated by *P. viticola* at the beginning of the infection, and increased by KPF to 10-fold expression (Figure 8B). The expression level slightly increased in KPF-treated leaves, followed by a sudden decrease. In the control group, the expression of *PR2* decreased slightly initially and then increased. In the control group, *PR4* expression increased during infection with a peak (35-fold increase) at 9 dpi (Figure 8C). *PR4* was greatly up-regulated with KPF treatment in the early stage of infection, reached a peak at 3 dpi with 60-, 30-, and 20- fold increases for 3%, 2%, and 1% KPF treatments, respectively. A rapid decrease occurred at 5 and 7 dpi, followed by a slight increase.

### The Direct Effect of Kaolin on *Plasmopara viticola* Sporangia

**FIGURE 9:**
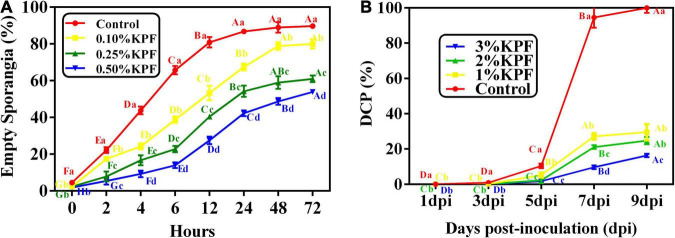
Effect of KPF on zoospore release and sporulation. Data represent the direct effect of KPF on *P. viticola* sporangia, including **(A)** empty sporangia (%), and **(B)** daily cumulative proportion of sporangia (DCP). For zoospore release assessment, sporangial suspension was mixed with water (Control), 0.1%, 0.25%, and 0.5% KPF. Then number of empty sporangia was estimated at 0, 2, 4, 6, 12, 24, 48, and 72 h for each treatment under the optical microscope. For DCP assessment, leaf discs were treated with sterile water (Control), 1%, 2%, 3% KPF and then were inoculated with a fresh *P. viticola* sporangia suspension (8 × 10^5^ sporangia.ml^– 1^), then the number of sporangia produced per cm^2^ was estimated at 1, 3, 5, 7, and 9 dpi. Three independent experiments with three biological repetitions each were carried out. Results correspond to the mean ± standard interval of three experiments, with three independent biological repetitions, with three technical repetitions each. According to Tukey’s test (*P* ≤ 0.05), means with the same lowercase letter are not significantly different among the different treatments for each time point, and means with the same uppercase letter are not significantly different among the different time points for each treatment.

The effect of various doses of KPF on percent empty sporangia was observed (Figure 9A). Zoospores were released at a very fast rate within 8h. Then the release gradually slowed down, followed by stabilization after 24h. Zoospore release was significantly reduced by KPF at any doses, compared to the water-treated control, especially in the first few hours. In general, the inhibition of zoospores release increased with increasing dosages of KPF. Inhibition of zoospores release was 10.83, 32.00, and 39.95% in water that was amended with 0.1, 0.25, and 0.5%KPF respectively according to the maximum percentage of zoospore release.

The effect of KPF on the daily cumulative proportion of sporangia was calculated every other day after infection until 9 dpi (Figure 9B). The sporangial production accumulated slowly before 5 dpi, and increased rapidly in the following 2 days, followed by stabilization at 8 and 9 dpi. The sporangial production was significantly suppressed to a great extent when KPF was pre-sprayed onto foliage. Maximum DCP was significantly decreased by KPF, though there was no significant difference between 1% and 2%KPF treatment.

## Discussion

This study aimed to evaluate the use of a KPF polymer to protect grape plants against downy mildew. KPF foliar application reduced grapevine downy mildew in the susceptible cv. Cabernet Sauvignon by severely limiting colonization of leaf tissues in leaf disc sporulation assays. To assess the mechanism of action, leaf discs were pretreated with KPF and inoculated with *P. viticola*, and then *in vitro* and in planta experiments were carried out.

### Kaolin Particle Film Protects Grapevine Leaves Against *Plasmopara viticola* and Reduces Oxidative Damage

Kaolin particle film treatment resulted in a good level of protection, with a significant reduction of *P. viticola* sporulation and disease index. Compared to the control plants, KPF-treated plants showed less and slower development of disease. This pattern is consistent with KPF protection observed in cucumber (Haggag, 2002). 60% protection could be provided by 3%KPF.

Sporangia collected from diseased leaves were used to infect the plants in this study. Uninucleate zoospores are released from mature germ-sporangium in water. When leaves are inoculated, zoospores germinate and form germ tubes that grow through stomata. Substomatal vesicles develop, followed by the formation of intercellular mycelium. At this time, many haustoria are formed and penetrate the cell walls of the mesophyll. Primary hypha can be observed (Burruano, 2000; Kiefer et al., 2002). Primary hypha was gradually observed in 1% and 2% KPF plants at 24 hpi and were not observed until 48 hpi in 3%KPF. However, the mycelium was already formed in the control and 1% KPF plants at this time, indicating that KPF delayed the formation of hyphae in leaves. As the infection progressed, the mycelium gradually increased and sporangiophores formed. Only a few hyphae were observed in the 2% and 3% KPF treatment, with less sporangiophores observed even in the final stage of infection. This indicates that fewer *P. viticola* invasion and slower pathogen development in plants that received high-dose KPF pretreatment. This lower infection was due to the lack of assessment to the entrance of the pathogen, because of the deposition of KPF. This observation of the infection progress shows that the response to *P. viticola* differs between control and KPF treatments. KPF application prevented infection and limited *P. viticola* development.

Stress conditions cause plants to accumulate excess reactive oxygen species (ROS). The over-generation of ROS leads to membrane lipid peroxidation, the destruction of membrane integrity, and other changes in physiological functions (Liu et al., 2015). In the control group, the levels of MDA increased during infection and the concentration of H_2_O_2_ was very high during early infection, indicating that *P. viticola* induced excess ROS in leaves. KPF treatment decreased the level of H_2_O_2_ and MDA throughout infection, indicating reduced damage caused by *P. viticola*.

In addition to be seen as highly damaging molecules for the plant, oxidizing agents also plays an important role in response for plant defense reaction against pathogen infection (Mittler et al., 2004). The generation of H_2_O_2_ has been considered as one of the earliest defense responses in the interaction between plants and pathogen (Alvarez et al., 1998). In this study, H_2_O_2_ accumulated rapidly in control group at the beginning of infection, followed by a slight increase, then decreased. But the increase of H_2_O_2_ in KPF treatment was later than in control. These results indicated the fewer and slower pathogen invasion due to the KPF application. However, although the overall content of H_2_O_2_ was lower, there was a greater extent of H_2_O_2_ increase in KPF treatments than in control, which indicate the benefit of KPF against biotrophic pathogen. This also made it possible that defense response of susceptible varieties could be activated by KPF application, at least in the Cabernet Sauvignon used in this study. After all, accumulation of H_2_O_2_ increase rapidly in resistant cultivar following inoculation, while little H_2_O_2_ generation is observed in susceptible cultivar (Liu et al., 2015).

### Kaolin Particle Film Prevents Direct Contact of Spores or Water With the Leaf Surface

Pathogen infection requires direct contact with the leaf surface. When a pathogen encounters an abaxial surface, zoospores invade through the stomata for infection (Vercesi et al., 1999). After the mycelium spreads within the leaf tissue, sporangiophores form and penetrate through the stoma, with the formation of a white downy mildew layer at the infected site. Sporangia are formed and zoospores are produced (Kiefer et al., 2002). Film-forming substances, such as chitosan, were shown previously to relieve pathogen attack by acting as barriers (El Hadrami et al., 2010; El Guilli et al., 2016). After KPF application, white particle residue remained on leaf surfaces after water evaporation, which forms a barrier. To test if KPF acted to prevent direct contact of the pathogen with the leaf surface, SEM was used to observe kaolin residues on leaves (Pedibhotla et al., 1999; Krzyzaniak et al., 2018). SEM observation revealed significant KPF residue on the leaf surface even after slight rinsing before observation. The presence of KPF can interfere with direct contact of the pathogens with the leaves. In addition, stomata were blocked by kaolin deposits, potentially blocking invasion of pathogens through the stomata. The SEM imaging confirmed that KPF application provides a physical barrier for leaves to hinder the invasion of pathogens. This also brings a limitation, that is the gas exchanges and photosynthesis may be affected due to the blocked stomata. Although it has been demonstrated to increase net photosynthesis by reflecting excessive light and reducing leaf temperature during summer stress (Campostrini et al., 2010; Dinis et al., 2018), it still needs more study in humid season that *P. viticola* is favoured to develop.

It’s interesting that a few deformed sporangia were observed in KPF treated leaf surface. This may be due to the insufficient cleaning of the leaf surface, even if the leaves were washed before SEM observation for visibility of the stoma. This provided a possibility for the hypothesis that KPF directly affects the morphology of sporangia. It is worth noting that the life cycle of oomycetes is short, and the pathogen is highly vulnerable between hatching from the sporangium and encystment at stomata (Troster et al., 2017). Therefore, kaolin may directly interfere with this pathogen, thereby reducing its aggressiveness.

### Kaolin Particle Film May Activities Defense Responses in Plants

In response to pathogen attack, there is over-production of ROS, leading to activation of anti-oxidative enzymes to scavenge these harmful substances (Limon-Pacheco and Gonsebatt, 2009). The activity of the antioxidant system increased in the early stage of biotic stress, indicating the activation of antioxidant defense followed by a slow decline (Kuzniak and Sklodowska, 2005). Many studies have found enhanced SOD, CAT, and POD activities in response to exogenous elicitors (Li et al., 2012; Prakongkha et al., 2013; Sood et al., 2013). In this way, oxidative damage can be mitigated. In this study, KPF treatment increased SOD, POD, and CAT activities compared to those in control leaves, thereby reducing reactive oxygen species. These results indicate that exogenous kaolin can activate plant defenses.

Kaolin particle film had other positive effects on the defense response. Pathogenesis-related (PR) protein acts in plant defense by damaging the structure of the pathogen (Selabuurlage et al., 1993; Giannakis et al., 2008). The activities of β-1, 3-glucanases (PR2) and chitinases (PR4) in grapevine leaves increase during pathogen response, with correlation between these two enzymes (Giannakis et al., 2008). Both β-1,3-glucans and chitin are major structural components of cell walls in higher fungi, and can be hydrolyzed by β-1,3-glucanases and chitinases, respectively (Selabuurlage et al., 1993). Therefore, fungal growth in a host can be inhibited by β-1,3-glucanases and chitinases. However, there is no chitin in the cell wall of oomycetes such as *P. viticola* (Maia et al., 2014). However, chitinase can still work by releasing some fragments which are material for weakening the pathogen cell wall (Yoshikawa et al., 1993; Selitrennikoff, 2001) to strengthen the host defense response. This study found increased activities of β-1,3-glucanases and chitinases in response to *P. viticola* attack. During infection, the activities of these two PR proteins increased first and then decreased. KPF treatment increased the activities of these two enzymes for all sampling dates, consistent with an effect of KPF to stimulate plant defense.

The production of phytoalexins is another important plant defense strategy that can reduce the severity of grapevine downy mildew. Secondary metabolites, such as resveratrol and flavonoids, are produced to reinforce the cell wall of host plants when under stress (Kuć, 1990; Poinssot et al., 2012). The accumulation of phytoalexin is regulated by *PAL*, which acts in the phenylpropanoid pathway (Sood et al., 2013). In this study, the activity of PAL was also improved by KPF, indicating that the stimulation of secondary metabolism is another way that KPF can increase resistance. The expression of *PR2*, *PR4* and *PAL* was up-regulated by *P. viticola* attack, and KPF also increased the expression of these three key genes during early infection compared to the levels in the control plants. This up-regulation is consistent with the observed increase in enzyme activity. These increases in resistance due to KPF application are further evidence that exogenous KPF can stimulate plant defense. Phytohormones play an important role in regulating plant resistance to various stresses (Glazebrook, 2005), and concentration changes in endogenous phytohormones indicate plant responses to stress. In this study, the levels of three important phytohormones (ABA, JA, and SA) were tested in different treatments during infection.

Abscisic acid plays a significant role in the post-infection phenomenon, and increased ABA levels causes stomatal closure and callose deposition to prevent pathogen invasion (Yasuda et al., 2008). There is a negative correlation between ABA and SA during the defense response. After infection, ABA levels do not change initially, and are much lower than SA levels in resistant cultivars (Kusajima et al., 2010). However, contradictory changes were observed in this study. During the infection period, both ABA and SA only increased slightly at 5 dpi, which is relatively late. The reason could likely be that Cabernet Sauvignon is a susceptible species. The pathogen suppresses the plant defense reaction and improves pathogen virulence by secreting phytotoxins and proteinaceous effectors into the plant cells (Boller and He, 2009), so the resistance function of susceptible varieties may be suppressed. KPF application increased ABA significantly and delayed the time point to reach maximum, indicating increased immunity. During the interaction between pathogens and plants, defense responses are triggered by local and systemic increases in endogenous SA levels. The increase in SA content is an essential modulator of the defense response, resulting in up-regulation of defense genes and production of PR protein (Gray, 2002). Thus, the accumulation of SA is positively correlated with the disease resistance of plants. In the resistant cultivar, early and high accumulation of SA was observed, with two rounds of SA accumulation observed after inoculation. However, the susceptible cultivar showed less of a change in SA levels (Liu et al., 2016). Considering that susceptible species were used in this study, it makes sense that the SA content of the control group did not increase significantly with infection. KPF increased the maximum SA content by nearly 3-fold, and made the peak time earlier. The increase in SA levels indicates that KPF plays a role in plant resistant improvement.

To further investigate the contribution of KPF to defense response improvement, the expression levels of key genes of pathogenesis-related (PR) proteins, activated by SA, were determined. SA is the most important plant signal molecule, and can be synthesized from phenylalanine ammonialyase (PAL) (Sawada et al., 2006) or from isochorismate (ICS) (Garcion et al., 2008). In this study, *PAL* exhibited a multi-fold (4- or 5-fold) increase in expression level in 2 and 3% KPF treatment samples at the early stage of inoculation, with later increases in expression levels in the control and 1% treatments. These expression patterns were consistent with SA content. The expression levels in leaves that received 2 and 3% KPF were not sustained throughout the whole infection process, leading to a decrease of SA content at the late stage of infection. However, the expression level of *ICS2* in all treatments showed little change during the infection process. This suggests a positive correlation between the activation of PAL and accumulation of SA in leaves after *P. viticola* infection, but no significant effect of *ICS2*.

The EDS1-PAD4 protein complex is an important activator upstream of SA synthesis (Lippok et al., 2007). After inoculation, expression of both *EDS1* and *PAD4* was initially induced rapidly in KPF-treated leaves (5- to 10-fold), but increased slowly in the control group. Interestingly, increased expression of *EDS1* and *PAD4* is not sufficient for resistance to downy mildew (Gao et al., 2014), suggesting that additional genes and proteins contribute to resistance. The enhancement of plant resistance to *P. viticola* due to KPF might act downstream of the signal pathway. The higher and faster expression induced by KPF treatment will induce a series of downstream defense responses to strengthen the resistance to pathogen invasion during early infection.

To test, the expression levels were measured for other defense-related genes. The expression of *NPR1* has been shown to be accompanied by the accumulation of SA and PR proteins (Malamy et al., 1990). *NPR1* interacts with the *TGA* class of transcription factors leading to the downstream expression of SA-dependent genes, thereby activating *PR* genes (Loake and Grant, 2007). Thus, the overexpression of *NPR1* plays an important role in strengthening grapevine defense against pathogen invasion. In this study, increased expression of *NPR1* was observed with KPF treatment at the early stage of infection, but later and lower expression was observed in the control group. Consistent with the pattern seen for *NPR1*, T*GA1* was also induced by KPF during the early stage of infection.

Further study of *PR* genes was carried out in this study. Production of PR protein is usually considered an important sign of plant resistance of pathogen invasion (Durrant and Dong, 2004). *PR* transcription and translation are highly elevated by pathogen attack (Kortekamp, 2006). The results obtained in this study showed that the expression of *PR1* in KPF treatment was obviously enhanced, and this effect persisted for the entire infection process. The enhanced expression of *PR2* and *PR4* due to KPF was more intense and rapid, but quickly dropped to a very low level. This indicated that KPF induced resistance in the early process of disease resistance, but this mechanism did not provide defense against *P. viticola* for a long period.

During pathogen-plant interaction, plant defense response can be activated by the pathogen. However, compared to resistant species, less SA is produced in susceptible species because of the low expression of *PAL* and *ICS2*, leading to low activation of plant defense reaction (Figueiredo et al., 2012). The slow gene activation of the control group observed in this study is consistent with this pattern. KPF application can activate the defense reaction. However, these results suggest that KPF induced high expression of many tested genes (*EDS1*, *PAD4*, *PAL*, *TGA1*, *NPR1*, *PR2*, and *PR4*) only appeared during the early stage of infection. Most were rapidly induced by KPF, peaked at 3 dpi, had a sharp decrease, and were then maintained at a low level. This pattern indicates that the activation of defense response by KPF does not last for a long period. Considering the effective prevention of *P. viticola* infection due to KPF application, the prevention of direct contact between pathogens and hosts and the early induction of host defense response must confer sufficient protection for the host. Fewer pathogens can invade KPF-treated hosts, suppressing early expansion. Therefore, defense responses are not required later.

Although JA-ethylene signaling transduction is generally considered to be associated with necrotrophic pathogens (Anderson et al., 2004), others reports demonstrate JA can act in resistance against *P. viticola* (Polesani et al., 2010). In this study, the JA content did not change much during the infection process, but exhibited a decreasing trend, suggesting that JA has little to do with resistance to *P. viticola*. Thus, although KPF slightly increased the concentration of JA, JA is not likely part of the resistance response due to the observed decrease during infection.

This evaluation of KPF exogenous application showed the elicitor efficiency of KPF to reinforce the defense of leaf discs. It may work through some mode of actions. First, the positive effects of elictors to stimulate plant defense responses have been demonstrated for silicon (Deepak et al., 2008; Yu et al., 2010; Nascimento et al., 2016; Farouk et al., 2017), aluminum (Massoud et al., 2012; Dufour et al., 2016), chitosan (Saberi and Panah, 2015), β-1,3-glucan sulfate (Trouvelot et al., 2008), melatonin (Sun et al., 2019), and some plant extracts (Boubakri et al., 2012; Krzyzaniak et al., 2018). These elicitors are usually used exogenously on leaves before or after infection to improve host defense against pathogens. Among these elicitors, the positive effects of silicon and aluminum on the stimulation of defense were considered in this study, since kaolin is composed of aluminosilicate. A possible direct influence of silicon should not be ignored. However, it is difficult to know how silicon and aluminum in kaolin penetrate inside the leaf tissues, and may not even be used by leaves due to its chemical inertness. Therefore, the hypothesis that kaolin induce plant resistance still needs a lot of studies.

It’s worth to be note that the elicitor efficiency of KPF is competitive compared with other inducers. For instance, cucumber seedlings pre-treated with melatonin showed about 40% reduction of disease index (Sun et al., 2019). And some plant extracts (Krzyzaniak et al., 2018) or plant-derived essential oil (Maia et al., 2014) also showed 70% and 40% protection, respectively. While other reported elicitors, such as silicon and bion, only provided 20% protection, chitosan provided protection similar to fungicides (about 50%) (Farouk et al., 2017). In this study, 65% maximum disease protection was achieved at a concentration of 3%KPF. This level of protection due to KPF is not lower than that provided by other inducers. Moreover, the production of KPF is easier and cheaper. Great advantage for KPF in the actual application process has been shown to encourage more research, and to facilitate it as a practice for crop management programs.

### Kaolin Particle Film Directly Act on *Plasmopara viticola* Sporangia

Zoospore release and sporulation are important parameters for *P. viticola* development. At the initial of the infection, zoospores encyst the stoma, and germ tube emerges and reaches into the substomatal cavity. Then substomatal vesicles develop with primary hyphae presence (Kiefer et al., 2002). The more zoospores are released, the more zoospores penetrate and encyst in the stoma, which result in serious infections and sporulation cycles. Zoospore release can be estimated based on the number of empty sporangia (Kiefer et al., 2002). To investigate that if KPF had direct effects on zoospores release, different dose of KPF were added to sporangial suspensions, and the empty sporangia were estimated in this study. No matter which time period is considered, the percentage of empty sporangia in KPF treatment are lower than that in Control. Moreover, the ANOVA revealed a significant effect of KPF on maximum zoospore release, though the dose of KPF is much smaller than in actual applications, in order to ensure a clear microscope field for observation. Therefore, fewer zoospores could encyst in stoma due to the KPF treatment, thereby reducing the disease severity.

Sporulation and sporangia accumulation can also be used to explain pathogen development. After the infection of zoospores, sporangiophores and sporangia are produced and form a dense, raised, white-cottony mildew on the underside of leaf. Sporangia release clonal zoospores as secondary inoculum (Pearson and Goheen, 1989). The sporulation and sporangia accumulation contributes a lot to the development and expansion of the infection. Therefore, the daily cumulative proportion of sporangia (DCP) were investigated in this study. At the beginning of the infection, the sporulation was extremely slow, no matter in which treatment. The sporangia accumulation of the control group began to accumulate obviously from the 4th day, and then it accumulated rapidly. However, the sporulation was significantly inhibited by KPF treatment. The accumulation of sporangia in KPF treatment was not only delayed, but also slow. These results suggested that KPF is a useful method for preventing sporangia accumulation, thereby limit the development and spread of downy mildew. The results of this study demonstrated that KPF can also directly act on pathogens and reduce their development, thereby limiting the development of downy mildew.

The positive effect of KPF in alleviating the development of downy mildew on cv. Cabernet Sauvignon leaves were demonstrated using *in vitro* inoculation assays. As shown in this study, KPF can utilize several possible mechanisms to protect against plant pathogens, including (i) direct blocking of spore contact with the leaf surface. The host is enveloped and the stomata are blocked by kaolin deposition, inhibiting the invasion and expansion of *P.viticola*; (ii) reduction of leaf damage. The accumulation of harmful substances, such as H_2_O_2_ and MDA, was reduced by KPF application; (iii) improved host defenses. KPF treatment increased the activities of antioxidant enzymes (SOD, POD, and CAT) and defense-related enzymes (PAL, chitinase, and β-1, 3-glucanase), which play important roles in plant defense. Plant defense-related reactions were activated by KPF, with increased expression of key genes and accumulation of SA. (iv) directly act on *P. viticola* sporangia. KPF reduced zoospore release and sporulation.

## Conclusion

In this study, the efficacy of KPF against grape downy mildew was demonstrated by *in vitro* inoculation assays. KPF acted both as a barrier and a resistance inducer to protect leaf tissues, and it can also directly act on sporangia by limiting the release of zoospores. This is only the first and a preliminary study of KPF on the alleviation of grape downy mildew. The efficacy of KPF against downy mildew should be confirmed on intact plants in field in the future. Additionally, further experiments should be performed to compare the efficacy of KPF and other conventional control agents (e.g., Bordeaux mixture). These questions provide more ideas for further research. They are very worthwhile to facilitate the incorporation of this novel, eco-friendly, and low-cost product into crop management strategies.

## Data Availability Statement

The original contributions presented in the study are included in the article/Supplementary Material, further inquiries can be directed to the corresponding author/s.

## Author Contributions

HL, HW, and YW conceived the project and designed the experiments. YW and XC performed the experiments with the help of YH, XH, and ZW. TX, QY, XD, and LZ provided technical assistance to YW. YW wrote the first draft of the manuscript. HL edited the manuscript with contributions from all authors.

## Conflict of Interest

The authors declare that the research was conducted in the absence of any commercial or financial relationships that could be construed as a potential conflict of interest.

## Publisher’s Note

All claims expressed in this article are solely those of the authors and do not necessarily represent those of their affiliated organizations, or those of the publisher, the editors and the reviewers. Any product that may be evaluated in this article, or claim that may be made by its manufacturer, is not guaranteed or endorsed by the publisher.
