# Supplementary figures and images for: Kaolin Particle Film Protects Grapevine cv. Cabernet Sauvignon Against Downy Mildew by Forming Particle Film at the Leaf Surface, Directly Acting on Sporangia and Inducing the Defense of the Plant
Source: Front Plant Sci. 2022 Jan 10;12:796545. doi: 10.3389/fpls.2021.796545 (PMC8784833; doi:10.3389/fpls.2021.796545)

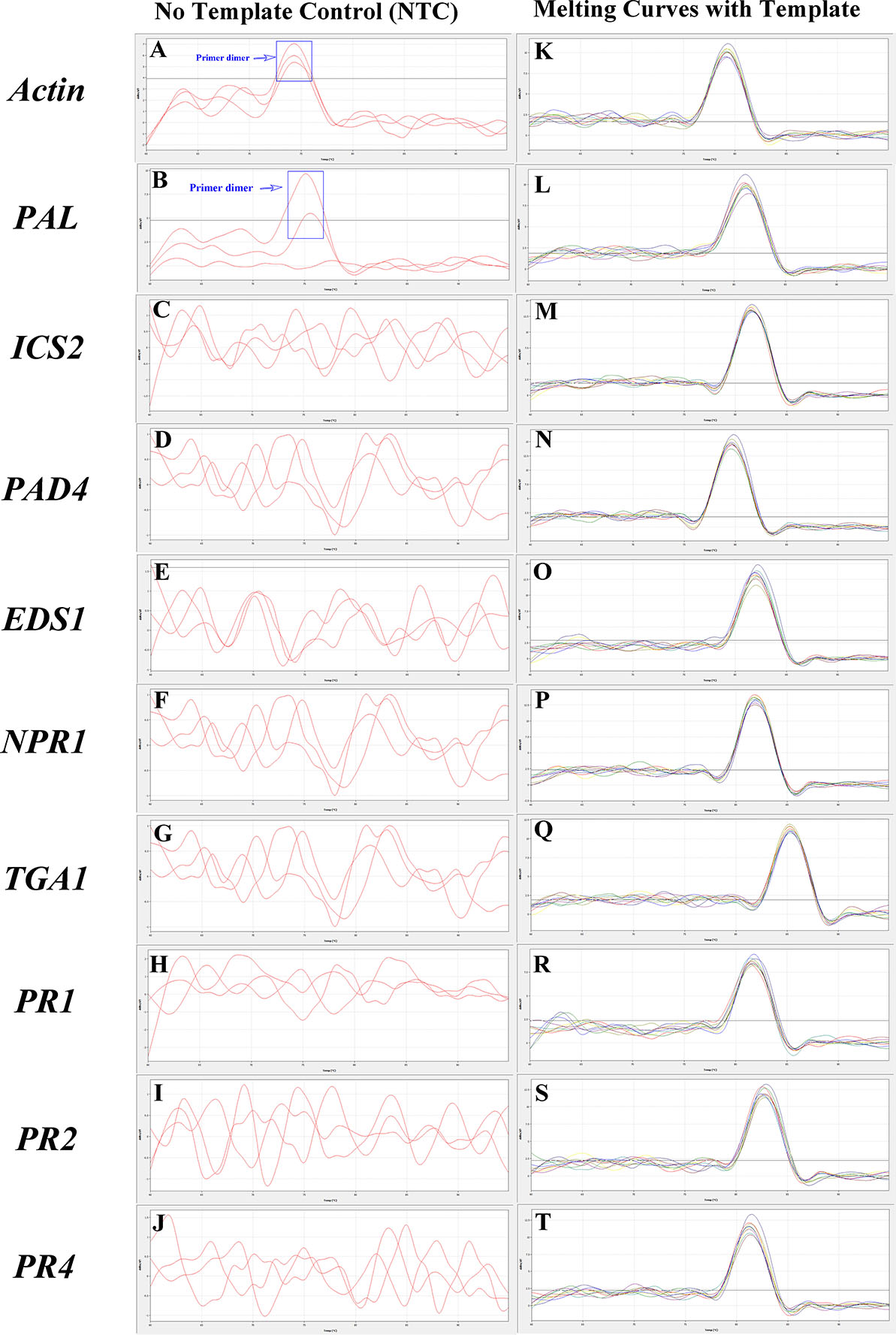

Supplement: Supplementary file 1 [file Image_1.JPEG]
